# How We Embedded Consumer Engagement Into Workplace Education and Continuing Professional Development: A Quality Improvement Initiative in a Tertiary Imaging Department

**DOI:** 10.1002/jmrs.70121

**Published:** 2026-09-02

**Authors:** Ilona Lavender, Mohamed K. Badawy

**Affiliations:** ^1^ Monash Radiology Monash Health Melbourne Victoria Australia; ^2^ Department of Medical Imaging and Radiation Sciences Monash University Melbourne Victoria Australia

**Keywords:** consumer participation, diagnostic imaging, patient‐centered care, professional education, radiography

## Abstract

Consumer engagement, meaning the active involvement of patients and carers with lived experience, is increasingly recognised as central to high‐quality, patient‐centred healthcare, and in Australia it is embedded in national accreditation standards. Within diagnostic imaging departments, however, consumer perspectives remain under‐represented in workplace education and continuing professional development (CPD), where educational activities have traditionally prioritised technical competency and workflow. This is despite the fact that clinicians frequently meet patients at vulnerable moments. This paper describes how a tertiary imaging department embedded consumer engagement across workplace education and CPD, governance and service improvement, and outlines practical lessons for departments wishing to implement similar initiatives. It draws on a series of initiatives undertaken within a tertiary imaging department between 2024 and 2025, including consumer storytelling within multidisciplinary workshops and continuing professional development, consumer co‐design of patient information resources, a dedicated patient‐experience role, and consumer participation in departmental governance. We considered these activities alongside established frameworks for patient involvement in health‐professions education and for consumer participation, and we reflected on the practical conditions that distinguish meaningful engagement from tokenism: clarity of purpose, attention to consumer wellbeing, and a shift from symbolic inclusion towards genuine partnership. The activities are illustrated with feedback volunteered by clinicians and consumers, although we are careful to note that these reflections are not a formal evaluation. We argue that consumer storytelling and structured engagement are feasible, low‐resource strategies that may promote reflection on empathy, communication and patient‐centred practice, while offering practical approaches for embedding consumer engagement within imaging departments. Formal evaluation of educational and patient outcomes remains the necessary next step.

## Graphical abstract

Consumer engagement is increasingly recognised as an essential component of patient‐centred healthcare, yet practical examples within diagnostic imaging remain limited. This article describes how a tertiary imaging department embedded consumer engagement through workplace education and continuing professional development (CPD), co‐design, governance and service improvement, and outlines practical lessons for departments wishing to implement similar initiatives.

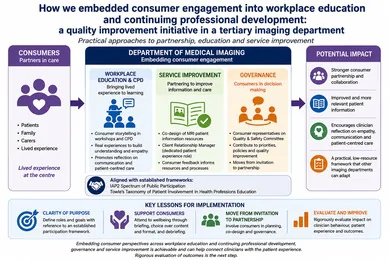

## 1 Introduction

Embedding consumer perspectives into workplace education and quality improvement is an emerging strategy for strengthening patient‐centred care within diagnostic imaging departments. Patient‐centred care is a defining domain of healthcare quality [1]. Shared decision making, in which patients are partners in their own care, is widely regarded as its fullest expression [2]. In Australia, partnering with consumers (patients, families and carers who contribute lived and living experience) is now a mandated accreditation standard that requires health services to involve consumers in the planning, design, delivery and evaluation of care [3]. In medical radiation sciences specifically, consumer engagement is increasingly applied to quality improvement and research [4].

Embedding consumer engagement is particularly important in diagnostic imaging. Clinicians often meet patients during emotionally challenging circumstances, including investigations for suspected cancer, acute stroke, pregnancy complications, chronic disease, traumatic injury, or other significant health events, where uncertainty and anxiety are common. Yet perceptions of what constitutes patient‐centred care differ between those who deliver imaging and those who receive it, and communication is a recurring point of difference [5]. Despite this, workplace education and continuing professional development within imaging departments have traditionally emphasised technical proficiency, image quality and throughput, with comparatively little structured attention to the patient experience [6].

Patient involvement in education is well established in medicine and nursing, where taxonomies have been developed and an emerging body of evidence describes potential benefits and practical approaches [7, 8]. Diagnostic imaging has been slower to adopt these approaches. Recent work signals growing momentum, including the formal evaluation of person‐centred care teaching in radiography curricula [9] and inclusive patient‐and‐public involvement in imaging research [10]. Even so, published examples of how departments practically embed consumer perspectives in education remain scarce. As Towle and colleagues observed some years ago, most patient‐involvement initiatives are isolated, one‐off events reported as basic descriptions, with little investigation of behavioural outcomes and persistent confusion over terminology [7]. This paper sets out to address that gap. We discuss the rationale for embedding consumer perspectives in workplace education and continuing professional development, namely, to strengthen patient‐centred care, empathy and communication by incorporating lived experience into workplace learning; describe a range of initiatives undertaken within our department as a worked example and draw out practical principles for departments wishing to do the same.

### 1.1 Defining Consumer Engagement

Terminology in this field is inconsistent, with a range of terms used to describe similar concepts, contributing to confusion within the literature [4, 7]. Throughout this article, we use the term *consumer* to include patients, family members and carers who contribute the perspective of lived experience. The terms *clinician* and *staff* include radiographers, sonographers, radiologists, nurses and allied health professionals involved in delivering health care services. Consumer engagement describes the active involvement of consumers in activities that shape education, services or governance. The International Association for Public Participation (IAP2) describes a spectrum of participation that ranges from informing and consulting through to involving, collaborating and empowering, with increasing levels of public influence over decision‐making [11]. Similar models of patient and public involvement in healthcare describe a progression from more passive or tokenistic participation towards meaningful partnership and empowerment [12].

### 1.2 Embedding Consumer Perspectives: A Departmental Example

Between 2024 and 2025, our Department of Medical Imaging, a single department operating across six hospitals within the Monash Health public health network, undertook several initiatives to embed consumer perspectives into workplace education, CPD and service improvement (Table 1A and Table 1B). These were developed under organisational priorities for patient‐centred care and consumer partnership, and were supported by consumer participation in departmental governance, including representation on committees.

**TABLE 1A:** Educational consumer engagement initiatives.

Initiative	Format	Participants	Consumer role	Purpose
Women's Health Imaging Workshop	Full‐day Saturday multidisciplinary CPD workshop (hybrid)	106 participants including radiographers, sonographers, radiologists, nurses and other healthcare professionals	Consumer representative with lived experience of breast cancer shared her experience of diagnosis and the imaging pathway (15–20 min presentation with facilitated discussion)	Increase understanding of the patient experience during breast imaging and diagnosis
Stroke Imaging Workshop	Full‐day Saturday multidisciplinary CPD workshop (hybrid)	89 participants	Stroke survivor shared personal experience of stroke diagnosis and imaging investigations (15–20 min presentation with facilitated discussion)	Improve understanding of the patient journey during acute stroke
Urology & Nephrology Imaging Workshop	Full‐day Saturday multidisciplinary CPD workshop (hybrid)	140 participants	Consumer shared personal experience of acute kidney injury and hospital admission (15–20 min presentation with facilitated discussion)	Highlight the emotional and psychological aspects of illness and diagnostic imaging
Departmental CPD education sessions	Discipline‐specific CPD sessions conducted during normal working hours	Monash Health radiographers and sonographers	Consumers shared lived experience during profession‐specific education sessions	Promote patient‐centred communication and reflection

**TABLE 1B:** Service improvement and governance engagement initiatives.

Initiative	Format	Participants	Consumer role	Purpose
MRI patient information posters	Consumer co‐design	Consumer review group	Consumers reviewed wording, readability, accessibility and usefulness before implementation	Improve clarity, accessibility and patient understanding of MRI information
Client Relationship Manager	Patient experience program	Patients, consumers and clinical teams	Patient feedback collected, analysed and translated into service improvement initiatives	Improve communication, patient information and overall patient experience
Radiology Quality and Safety Committee	Departmental governance	Consumer representative and multidisciplinary committee members	Consumer representative contributed lived‐experience perspectives to discussions on patient information, quality activities and service improvement	Embed the consumer voice into departmental governance and decision‐making

*Note:* All multidisciplinary workshops were held on Saturdays at a single host site in Melbourne, Australia, and delivered in a hybrid format. Approximately half of attendees were from the Department of Medical Imaging within Monash Health, with the remaining participants joining from external healthcare organisations.

Abbreviation: CPD, continuing professional development.

Although these initiatives were developed pragmatically to meet local departmental needs, they align with established frameworks for patient involvement in health professions education and consumer participation. Consumer storytelling within workshops primarily reflected the “involve” level of the IAP2 Spectrum, while consumer co‐design of patient information resources and participation in departmental governance aligned more closely with “collaborate”. Similarly, Towle's taxonomy informed our progression from consumers contributing lived experience within educational activities towards sustained partnership through co‐design and governance.

Between 2024 and 2025, the department delivered three multidisciplinary imaging workshops as part of its continuing professional development (CPD) program. The workshops were held on Saturdays at a single host site in Melbourne, Victoria, Australia, and delivered in a hybrid format, allowing participants to attend either in person or virtually. Approximately half of the attendees were from Monash Health, with the remaining participants joining from other healthcare organisations. A total of 335 participants attended the three workshops (106 attendees at the women's health workshop, 89 at the stroke workshop and 140 at the urology and nephrology workshop). Workshops were attended by radiographers, sonographers, radiologists, nurses and other healthcare professionals, with attendance contributing to CPD requirements for the relevant professions. Each workshop focused on a clinical theme and combined presentations across multiple imaging modalities with a 15–20 min consumer presentation followed by facilitated discussion.

Consumers were invited to share their experiences of healthcare and diagnostic and therapeutic imaging within two educational formats. The first comprised three multidisciplinary imaging workshops described above. The second comprised regular discipline‐specific CPD sessions conducted during normal working hours for Monash Health radiographers, sonographers and other imaging staff. An internally developed hospital platform enabled participants to submit questions anonymously via QR codes throughout each workshop. The consumer presentations were intended to provide the human context of imaging alongside the technical content. Consumers were identified through existing departmental relationships rather than open recruitment. One consumer was identified through her role as a consumer representative on the department's Radiology Quality and Safety Committee. Given her lived experience of breast cancer, she was invited to share her experience at the Women's Health Imaging Workshop. A stroke survivor was invited by the clinician who had treated him, and a consumer who had experienced acute kidney injury was invited through the renal nursing team.

Attention to consumer wellbeing was built into the process, given that recounting personal healthcare experiences can be emotionally demanding. Before each session, consumers participated in a briefing to explain what to expect, discuss the workshop format and provide reassurance about the process. They were offered suggestions for what they might cover but were free to choose their own focus, to add anything they wished, and to leave out anything they preferred not to discuss. They were told that they could pause, finish or leave at any point, and that they could decline to answer questions. Consumers also chose the format that suited them, whether to speak and then take questions, to talk freely, or to follow a more structured approach. After each presentation, every consumer was contacted by email, and a check‐in was offered.

Consumers also contributed to service improvement. They reviewed draft patient information posters for the MRI waiting area, which explained MRI safety requirements and appropriate clothing. They commented on clarity, readability and usefulness from a patient perspective, prompting revisions to simplify the language, improve the layout, clarify clothing requirements before MRI examinations, and enhance the accessibility of the information before the revised posters were displayed. Following implementation, the revised posters received positive feedback from patients and staff, and the success of this initiative led to the development of additional consumer‐informed patient information posters within the department. This reflects a co‐design approach that has been shown to improve the accessibility of patient information resources in other clinical settings including endoscopy services [13].

The department supports consumer engagement in two further ways. A full‐time (1.0 EFT) Client Relationship Manager, who is a Certified Patient Experience Professional, works across all six hospital sites within the health network. They systematically collect patient feedback, identify recurring themes and work with clinical teams to implement service improvements. Examples include reviewing patient compliments and complaints, identifying opportunities to improve communication and patient information, and working collaboratively with clinical teams to implement changes that enhance the patient experience. Consumers are also represented on the department's Radiology Quality and Safety Committee, where they contribute lived‐experience perspectives to discussions on patient information resources, service improvement initiatives, quality activities, and patient experience issues relevant to imaging services.

### 1.3 What We Observed

Across the three multidisciplinary workshops, 335 participants, including radiographers, sonographers, radiologists, nurses and other healthcare professionals, attended sessions that included consumer storytelling (Table 2). Participants were invited to complete a voluntary post‐event satisfaction survey following each workshop. The survey was designed to evaluate the workshop overall and included questions relating to the quality and relevance of the presentations, workshop organisation, venue and catering, together with an optional free‐text section where participants could provide any additional comments. It was not designed to specifically evaluate the consumer engagement component. Nonetheless, several participants chose to comment on the consumer presentations in the unsolicited free‐text responses. These comments are included to illustrate participants' reflections on the consumer engagement initiatives rather than to represent the findings of a formal evaluation, a limitation that characterises much of this field [7].

**TABLE 2:** Attendance and post‐event survey responses across the three multidisciplinary workshops.

Workshop	Attendees	Responses	rate
Women's Health	106	22	20.8%
Stroke	89	60	67.4%
Urology & Nephrology	140	19	13.6%
**Total**	**335**	**101**	**30.1%**

Within those limits, several clinicians chose to comment on the consumer contributions. One wrote, “Great hearing the personal experience of a patient affected by AKI. I'm more aware of their emotional needs and will make it a priority over the pressure of time constraints.” Another reflected, “Loved the presentation by the consumer. It reminds us all why we do this work.” A third described a highlight as “listening to the stroke survivor speaking about his experience.” These comments echo a recurring observation in the wider literature, that hearing directly from patients prompts clinicians to reflect on the emotional dimensions of care and on their own communication [8].

Consumers, in turn, provided informal feedback following the workshops and described their participation as meaningful. They spoke of gratitude for the care they had received, a wish to give back, and a desire to be a voice for others at vulnerable times. As one consumer advisor put it, “We are humans, not just another scan.” Guest consumers valued the opportunity to contribute, with one noting that it was “an honour to be able to share my story.” The exchange appeared mutually beneficial, with consumers feeling heard and staff reconnecting with the purpose of their work. Several consumers also expressed appreciation that their feedback resulted in tangible changes to patient information resources and educational activities, reinforcing that their involvement had a meaningful impact.

### 1.4 From Participation to Partnership: Avoiding Tokenism

Inviting consumers to share their stories carries a responsibility to do so well. Without a clear and shared purpose, consumer involvement can become tokenistic, offering symbolic inclusion that reaffirms existing practice rather than genuinely informing it [12, 14]. Tokenism has been characterised by unequal power, limited impact, and involvement that serves the organisation's purpose rather than the consumers [12, 14]. It maps onto the lower levels of participation frameworks, where consumers are informed or consulted but do not shape decisions [11, 15]. Towle's taxonomy draws a similar distinction between one‐off contribution and sustained partnership in which consumers help set the agenda [7]. Reflecting on our initiatives against the IAP2 Spectrum, we consider our current approach to sit predominantly between ‘involve’ and ‘collaborate’. Consumer storytelling primarily represents involvement, whereas co‐design of patient information resources and consumer participation in departmental governance demonstrate greater collaboration and influence over departmental activities. Experiential learning theory offers a complementary explanation for why first‐hand narrative, when it is reflected upon, can change practice in ways that didactic content alone may not [16].

In our experience, three conditions mattered. The first was clarity of purpose, so that consumer storytelling was intentionally aligned with patient‐centred goals by promoting person‐centred communication, valuing consumers' lived experiences, supporting partnerships in care, and informing improvements to healthcare delivery [3]. The second was attention to consumer wellbeing, supported through briefing, choice over content and format, and a check‐in afterwards. The third was a shift from invitation towards partnership, which was most evident where consumers contributed through governance and co‐design rather than as speakers alone. Consumers also described their participation as meaningful, particularly where they could see that their feedback had informed tangible improvements to patient information resources, educational activities and departmental practice. A practical next step which we are now adopting is to involve consumers in planning workshops and not only in delivering them.

### 1.5 Recommendations for Practice

Departments seeking to embed consumer perspectives in workplace education, continuing professional development and service improvement may find the following useful. First, define consumer roles and the purpose of their involvement with reference to an established participation framework, such as the IAP2 Spectrum or Towle's taxonomy of patient involvement in health professions education [7, 11]. Second, identify consumers through existing consumer committees, treating clinicians or partner services, and formalise briefing, support and debriefing to protect wellbeing. Third, obtain consent for stories to be shared and, where relevant, published. Fourth, extend engagement beyond storytelling to the co‐design of patient materials and to governance. Fifth, plan evaluation from the outset, including structured evaluation of the consumer experience of participation alongside participant learning and, where feasible, consumer‐led co‐evaluation.

### 1.6 Limitations and Future Directions

This report describes the experience of a single Department of Medical Imaging operating across six hospitals within one Australian public health network. Although the workshops were open to external participants, the initiatives were implemented under a single departmental governance structure. The reflections we report comprise spontaneous free‐text comments from workshop attendees and informal feedback from participating consumers rather than a purpose‐designed evaluation. Response rates to the post‐event surveys were also low and uneven, ranging from 13.6% to 67.4% across the three workshops, so the comments reported here may be subject to non‐response bias and cannot be assumed to represent the views of all attendees. Consequently, they cannot demonstrate impact on clinician behaviour or patient outcomes, which is the gap identified across this field two decades ago and still only partially addressed [7]. Addressing it is the clearest priority for future work. A formal mixed‐methods evaluation would substantially strengthen the evidence base, using a survey designed to assess the consumer component, validated empathy or attitudinal measures, and systematic qualitative analysis of solicited responses. Consumer‐led co‐evaluation of the initiatives would add further value.

## 2 Conclusion

Embedding consumer perspectives in workplace education and CPD within imaging departments is a feasible and low‐resource approach that may play an important role in connecting clinicians with the patient experience and fostering reflection on the empathy, communication and patient‐centred care required for high‐quality service delivery. Consumer engagement can operate at several levels, including storytelling in education, co‐design of patient information resources and participation in governance. Rigorous evaluation of educational, organisational and patient outcomes remains the necessary next step.

## Funding

The authors have nothing to report.

## Ethics Statement

This work was undertaken as a quality improvement activity and received Monash Health Quality Assurance approval (QA/127535/MonH‐2026‐525,239(v1)).

## Conflicts of Interest

The authors declare no conflicts of interest.

## Data Availability

The data that support the findings of this study are available on request from the corresponding author. The data are not publicly available due to privacy or ethical restrictions.
